# Cancer patients and COVID-19 vaccination, from safety to protocol adherence: A real-life setting report

**DOI:** 10.3389/fonc.2022.1014786

**Published:** 2022-10-03

**Authors:** Haitam Lamtai, Saber Boutayeb, Hind Mrabti, Ibrahim El Ghissassi, Hassan Errihani

**Affiliations:** Department of Medical Oncology, National Institute of Oncology, University Mohammed V, Rabat, Morocco

**Keywords:** COVID-19 vaccines, cancer, immunocompromised patient, side effects, drug therapy

## Abstract

**Background:**

The SARS-CoV-2 pandemic has slowed down cancer prevention and treatment strategies; consequently, cancer patients are prioritized to get the COVID-19 vaccines. Being constantly threatened by a new outbreak, the dive within the immunogenicity response is of great value; nonetheless, evaluating the side effects of these vaccines on fragile patients will assure their adherence to the vaccination protocol.

**Objectives:**

This study sets out to investigate the adverse events reported about the vaccine according to its doses and types, and to compare the prevalence and severity of toxicities across two subgroups of cancer patients, those who received the injection during active therapy cycles, and those who have not started the therapy yet at vaccination time, moreover, this paper examines the will and commitment of this population to the vaccination schemes.

**Methods:**

This is an observational, retrospective, cohort study, in which we conducted a semi-constructed interview with 415 random solid cancer patients treated at the National Institute of Oncology in Morocco. The assessment of adverse events was carried out with a standardized scale.

**Results:**

Eleven months after the launch of the campaign, 75.2% of patients received at least one dose of the vaccine. Altogether, the analysis demonstrates a significant difference between the adverse effects reported post the second dose compared to the first one (p=0.004; odds ratio=2 [95% CI: 1.23 - 3.31]). Besides, the results indicate an increase in the rank of the severity of systemic events (p<0.001, r=0.28) after the second dose, but not for the local events (p=0.92, r=0.005). In the adjusted subgroup analysis, no effect was detected linking active therapy with the occurrence of toxicity (p=0.51, v=0.04) as well as with the level of severity reported after both; the first and second dose. Due to the fear of interactions with the therapy, we noticed a significant trend to delay the booster dose among the participants who completed the initial vaccine protocol.

**Conclusion:**

A considerable body of evidence exists to persuade cancer patients to take the Coronavirus vaccines, and to also follow their vaccination schemes under the supervision of their treating physicians.

## Introduction

The World Health Organization declared the SARS-CoV-2 pandemic on March 11, 2020. A succession of events led the world, especially the third world and developing countries, to a rather fatal, and global health crisis. Cancer patients were markedly affected; the delay in disease screening and therapeutic strategies directly impacted the programs planned by various actors (1). To alleviate this burden, prophylactic strategies were required; consequently, the WHO have allowed an Emergency Authorization for coronavirus vaccines based on interim results.

As part of the program that aims to regulate the spread of the virus, the Moroccan scientific committee, approved for emergency use on January 28, 2021: 1 inactivated virus vaccine (Sinopharm/BBIBP-CorV), 1 mRNA vaccine (Pfizer-BioNTech/BNT162b2), and 2 adenovirus-based-vaccines (Oxford–AstraZeneca/ChAdOx1 nCoV-19, and Johnson & Johnson/Ad26.COV2.S). Since many studies pointed out the increasing risk of mortality and severity in immunocompromised individuals compared to the general population, the National Comprehensive Cancer Network and the European Society for Medical Oncology, endorsed the policy that tumor patients need to be prioritized in the vaccine campaign (2), and accordingly published their guidelines to ease the vaccination process (3). However, patients with comorbidities; especially cancer patients, are still hesitant to initiate the standard scheme (4, 5).

Cancer induces a chronic immune response in the organism which results in T cell exhaustion (6). Accordingly, we have to ensure that the vaccination will not cause more T-cell depletion. Furthermore, systemic inflammation after contact with SARS-CoV-2 leads to an increase in inflammatory cytokines, such as IL-6, IL-8 and TNF-α, that may intensify treatment resistance and tumor growth (7). With that in mind, the administration of a live or attenuated COVID virus vaccine could be dangerous. Another critical point is the predisposition of cancer patients to develop cancer-associated thrombosis initiated by the tissue factor and the interim reports concerning the occurrence of blood clots after COVID immunization (8–10).

Numerous publications in the literature focused on one kind of vaccine, however, in practice, the challenge of herd immunization has exposed the population to a wide variety of heterogenic vaccines; therefore, and by providing a pooled analysis of the adverse events reported by solid cancer patients and evaluating their level of adherence to the vaccination protocol; we conducted a study that helps to fill in the research gaps and contributes to the public health field.

## Materials and methods

### Study design and participants

This observational retrospective, cohort study, had been conducted between November 22, 2021 and January 31, 2022, at the National Institute of Oncology-Rabat, one of the leading oncology centers in Morocco (11). Patients who have been visiting the Oncology Day Hospital to receive their therapy were recruited. The inclusion criteria were: (i) giving informed consent; (ii) patients aged 18 years and older (iii); a confirmed diagnosis of solid tumors (iv); patients with a history of sars-cov-2 infection will be also recruited (v); patients who received the flu vaccine will be eligible.

Patients who did not meet the aforementioned inclusion criteria were excluded from this study. After a review of the existing literature, a sample size of 400 or more has been suggested as adequate for studying our population.

The study received the approval from the biomedical ethics committee of the faculty of medicine in Rabat: CERB (Identifier: P bis-22).

### Data collection

An investigator randomly enrolled patients who visited the daycare unit to receive their therapy. After the patient’s informed consent, data collection was conducted using a semi-constructed interview. The investigator asked targeted and specific kinds of questions in order to fill in a thorough questionnaire. In the end, a debriefing with each individual followed, to inform them about the latest findings of the recent studies.

The patients were asked about their clinical background, previous SARS-CoV-2 infection, and its severity level. The type and date of the vaccination were checked from the vaccination pass. All vaccinated patients were asked detailed adverse events questions concerning the first, second, and third immunizations, which we adapted from the “Guidelines for Classification Standards of Adverse Events in Clinical Trials of Prophylactic Vaccines” (12). Adverse events were classified from mild to severe: mild; do not interfere with activity, moderate; interfere with activity, severe; prevent daily activity, and finally life-threatening. The severity of some adverse events reported that involved precise measures (for example, fever), couldn’t be obtained retrospectively and were therefore considered as mild in our analysis. The unvaccinated patients, those who delayed the standard scheme, and the ones who didn’t want to receive the booster dose, were asked about the reasons behind their decision. The following information was gathered from patients’ electronic health records: cancer type and stage, date of cancer diagnosis, treatment phase and therapeutic strategies.

Data were exported to the Research Electronic Data Capture (REDCap) application, a software system for electronic secure data capture: This platform is hosted by the Cancer Research Institute - Fez - Morocco (IRC) (13). Registries were de-identified before the statistical analysis.

### Outcomes

The co-primary endpoints of this study were to compare the prevalence and severity of adverse events for each vaccine dose in this population, and to explore the toxicity in two subgroups of patients: Cancer patients who received the vaccine during active therapy, and those who have not started the therapy yet during the vaccination period.

Other secondary endpoints were explored; for instance: The relation between the type of vaccine and the occurrence of adverse events, the association between clinical characteristics and hesitancy of cancer patients who didn’t complete the three doses scheme, as well as those who were unvaccinated.

### Statistical analyses

We report categorical variables as counts (n) and percentages (%). The variables are reported using means and standard deviations (SD) or medians and interquartile ranges (IQR) depending on the distribution normality.

We utilized the R program (Version 4.1.0), to compare the paired binomial data of the symptoms declared after each dose using the McNemar test, with the exact2x2 package and the mid-p method as it controls better for the type I error rate (14). The Pearson’s Chi-squared and Fisher’s exact tests were performed to compare categorical variables between groups with the functions chisq.test() and fisher.test(). And the difference between proportions tests, if assumptions were to meet, was implemented using prop.test().

Using the software IBM SPSS statistics (Version 26.0.0): A Wilcoxon signed-rank test was applied to compare the level of severity of local and systemic effects reported, for those who responded to both the first and second dose questions. With the Mann-Whitney U test, we explored, in the adjusted group of participants vaccinated after the cancer diagnosis, the difference between the severity level of toxicity reported in the subgroup who received the vaccine shot, while on active therapy in comparison to the one who hadn’t started the therapy yet.

All tests were 2-sided with a 5% type I error. The association measures: odds ratios (OR); effect sizes (r); and Cramer’s V (v) are reported and no corrections were made for multiple comparisons.

## Results

In our study, 417 patients were approached, one refused to participate, and one had hematologic cancer. Altogether 415 participants with solid malignancies were enrolled *via* an informed-consent process (**Table 1** **)**.

**Table 1:** Baseline characteristics of the population.

	Vaccinated after cancer dg (n=197)	Vaccinated before cancer dg (n=115)	Not vaccinated (n=103)
**Median age, years *(IQR)*** **Age range, n (%)** **<40 years** **40-60 years** **>60 years**	57 (49-68) 14 (7.1) 99 (50.3) 84 (42.6)	59 (49-66) 6 (5.2) 59 (51.3) 50 (43.5)	51 (42-60) 22 (21.4) 57 (55.3) 24 (23.3)
**Sex, n (%)** **Male** **Female**	74 (37.6) 123 (62.4)	39 (33.9) 76 (66.1)	26 (25.2) 77 (74.8)
**Height in meters (SD)**	1.64 (0.08)	1.63 (0.09)	1.63 (0.08)
**Weight in kilograms (SD)**	65 (12.35)	66 (14.08)	65.8 (15.8)
**CCI**^a^ *(IQR):*	7 (4-8)	6 (3-8)	6 (3-8)
**Non-oncological comorbidities, n (%)** ***Cardiovascular disease^b^*** ***Chronic lung disease*** ***Diabetes mellitus*** ***Others^c^***	28 (14.3) 36 (18.3) 16 (8) 11 (5.5)	33 (28.7) 23 (20) 21 (18.3) 12 (10.5)	20 (19.4) 13 (12.6) 14 (13.6) 7 (6.8)
**Previous COVID-19 infections, n (%)**	25 (12.7)	13 (11.3)	14 (13.6)
**Malignancies**^d^, n (%): ***Breast*** ***Gynecological*** ***Genito-urinary tract*** ***Gastrointestinal*** ***Thoracic*** ***Head and neck \ Brain*** ***Skin*** ***Bone***	76 (38.6) 26 (13.2) 24 (12.2) 49 (24.9) 14 (7.1) 4 (2) 1 (0.5) 3 (1.5)	44 (38.3) 16 (13.9) 2 (1.7) 33 (28.7) 17 (14.8) 2 (1.7) 1 (0.9) 0	43 (41.7) 13 (12.6) 6 (5.8) 28 (27.2) 6 (5.8) 5 (4.9) 1 (1) 1 (1)
**TNM staging, n (%)** **I** **II** **III** **IV** **Missing data**	7 (3.6%) 26 (13.2%) 52 (26.4%) 111 (56.3%) 1 (0.5%)	7 (6%) 31 (27%) 27 (23.5%) 50 (43.5%) -	2 (2%) 14 (13.6%) 30 (29.1%) 57 (55.3%) -

a Charlson comorbidity index.

b Congestive heart failure, peripheral vascular disease, hypertension.

c Others: dementia, systemic disease, peptic ulcer disease, liver and kidney disease, hemiplegia, cerebrovascular disease and AIDS.

d Breast cancers for males and females. Gynecological cancers include ovarian, cervical, endometrial and vulvar cancers. Genitourinary cancers: prostate, bladder and kidney cancers.Gastrointestinal cancers: tumors of the colon, rectum, stomach, pancreas, esophagus, anus, gallbladder, liver, and bile duct. Thoracic cancers: lung and mediastinal tumors. Head and neck\brain cancers: larynx, pharynx and mouth and 1 case of glioblastoma.

In the vaccinated cohort, 312 (75.2%) patients had received at least one shot of the vaccine. The median age of the vaccinated cancer population was 58 years *(IQR, 49-67 years)*, and 199 (63.8%) were female. For the body mass index, the median was 24 kg/m² *(IQR 20.75-27.2)*. The Eastern Cooperative Oncology Group performance status scale was superior to 1 in 14.4% of the cases. Concerning the comorbidities, the calculated median of the Charlson Comorbidity Index (15) was 6 *(range 2-12)* and 16.7% of the vaccinated patients were treated for hypertension (**Supplementary Table S1**).

**Figure 1:**
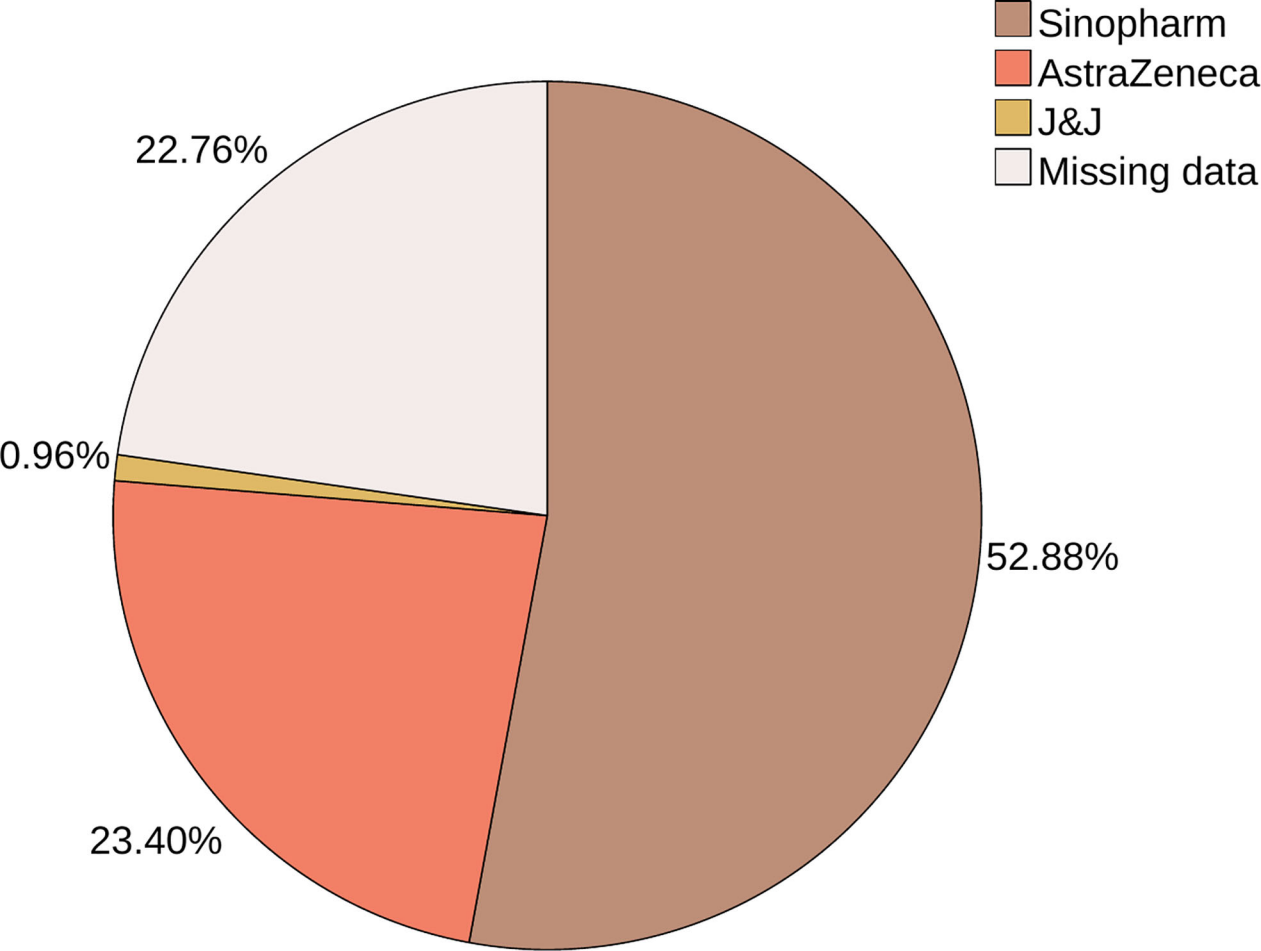
The vaccines used for the standard scheme.

Regarding other vaccinations, only two participants have taken the seasonal flu vaccine. In the initial protocol, more than half of our population was vaccinated with the Sinopharm vaccine, followed by the AstraZeneca vaccine, and then the Johnson & Johnson vaccine. For 71 patients, the information about the type couldn’t be verified (**Figure 1**). Thirteen patients didn’t complete or delayed the standard scheme of two doses: ten because of personal or medical decisions following the cancer diagnosis and three due to side effects after the first dose, namely severe fatigue, joint pain and myalgia. Nineteen (6.4%) patients that completed the standard protocol have received the booster dose. The brands’ frequencies for the booster shots were as follows: Sinopharm (42.1%), AstraZeneca (5.3%), and Pfizer (5.3%).

A cohort with solid malignancies was exclusively included in this study, with breast cancer being the most prevalent (39.3%). The second most common type of cancer was gastrointestinal cancer (26.5%), followed by gynecological cancers (13.3%), thoracic malignancies (8.9%), genitourinary tract (7.7%), head and neck cancers (2.7%), bone (1%), and finally skin cancers (0.7%). Most of the participants had metastatic growth (56%), and were diagnosed less than 12 months (65%) from enrollment.

The cohort diagnosed less than 12 months from study enrollment; tended to refuse the vaccination while receiving cancer medication **(** **Table 2** **)**. The median number of days between the vaccine and the cancer therapy was 15 days *(IQR 10 to 15)*. We noted that the group who were under active therapy did not consult the treating physician before vaccination 37% of the time.

**Table 2:** Characteristics of patients fully vaccinated while on active therapy with the unvaccinated patients.

	Vaccinated while on active therapy (n=103)	Not Vaccinated (n=103)	P-value
**Time from cancer diagnosis to study enrollment (%)** ** <12 months** ** ≥12 months**	28.1 71.9	60.2 39.8	**<0.001**
**Malignancies (%)** ** Breast** ** Gynecological** ** Genito-urinary tract** ** Gastrointestinal** ** Thoracic** ** Head and neck \ Brain** ** Skin** ** Bone**	39.8 13.6 12.6 22.3 7.8 1.9 1 1	41.7 12.6 5.8 27.2 5.8 4.9 1 1	**0.66**
**Metastatic cancer (%)**	72.8	59.2	**0.05**
**Therapeutic strategy (%)** ** Adjuvant** ** Neoadjuvant** ** Palliative**	13.6 11.6 74.8	25.3 19.4 55.3	**0.01**
**Therapy already received (%)** ** Chemotherapy**^e^ alone ** Chemotherapy+ Radiotherapy +/- Hormonotherapy** ^f^ ** Chemotherapy + Targeted Therapy** ^g^ ** Chemotherapy + Hormonotherapy** ** Hormonotherapy +/- Targeted Therapy**	39.8 26.2 17.5 12.5 4	60.3 19.4 8.7 8.7 2.9	**0.05**
**Corticosteroids (%)**	8.7	4.9	**0.40**

Chemotherapy used: Antimetabolites: Methotrexate - Fluorouracil - Capecitabine - Gemcitabine - Etoposide; Spindle toxins: Vincristine – Docetaxel – Paclitaxel - Vinorelbine; Alkylating agents: Cyclophosphamide – Ifosfamide – Irinotecan; Platinium-based agents: Carboplatin – Oxaliplatin – Cisplatin; Cytotoxic antibiotics: Epirubicin - Bleomycin – Doxorubicin.

Hormonotherapy: Tamoxifen – Bicalutamide – Exemestane – Anastrozole – Fulvestrant.

Targeted Therapy: Bevacizumab – Palbociclib – Panitumumab – Trastuzumab – Pertuzumab – Cetuximab - Rituximab.

In our cancer population, 52 (12.5%) have already tested positive for the SARS−CoV−2, 36 (69.2%) before vaccination and 15 (28.8%) after immunization (OR = 0.14 [95%CI 0.065-0.26]), and one patient tested positive twice before and after vaccination.

**Figure 2:**
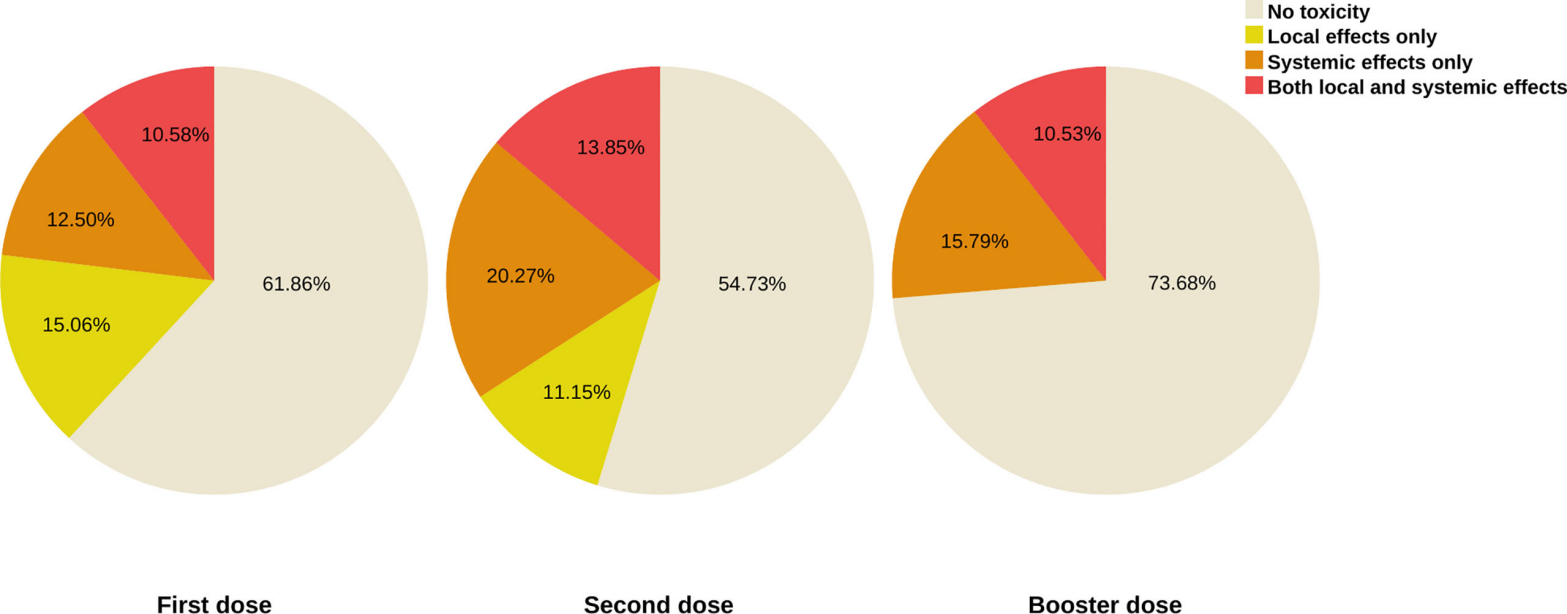
Type of toxicities reported after each dose in our cohort.

Using the Mid-p McNemar test; we analyzed the consistency in responses of 296 patients who answered questions about the symptoms after the first and second doses for two levels, those who reported no toxicity, and those who declared any local or systemic toxicity (**Figure 2**). A significant difference between the two doses was detected (p = 0.004; OR = 2 [95% CI: 1.23-3.31]) (**Supplementary Table S2**).

In a further analysis with the Wilcoxon signed-rank test; we compared paired reports about the severity grade of adverse events after the first, and second doses. Our findings outlined a significant change in the severity rating of systemic effects declared (p<0.001, r = 0.28) but no significant variability in local effects (p = 0.92, r = 0.005). Precisely, the frequent local side effect after the second dose in our population was pain at the injection site (16.9%, n=70), followed by both swelling (1.7%, n=7) and lymphadenopathy (1.4%, n=6). Concerning the systemic toxicity, fatigue (15.2%, n=63) and fever (11.6%, n=48) in the days following vaccination were the most reported, next, joint pain (2.7%, n=11), myalgia (2.2%, n=9), and four cases (1%) of chest pain following the 2nd dose were reported. Ultimately, one case (0.2%) of generalized pruritus persisting for months after the second vaccination was described. A subsequent analysis found no evidence of an association between the two frequent types of vaccine and the occurrence of toxicities (**Table 3**).

**Table 3:** Side effects reported after the first and second vaccine doses for the two frequent vaccines in our population.

	First dose		Second dose		P-value*
	AstraZeneca	Sinopharm	AstraZeneca	Sinopharm	
Local toxicity in %
Pain	23.3	24.8	24.7	20.4	**0.48**
Erythema	1.4	1.2	0	2.4	**0.31**
Swelling	1.4	2.4	2.7	2.4	**1**
Pruritus	0	1.2	0	1.2	**1**
Lymphadenopathy	0	0.6	2.7	1.8	**0.64**
Systemic toxicity in %
Vomiting	2.7	0.6	0	0	**1**
Nausea	1.4	0.6	1.4	0.6	**0.52**
Diarrhea	0	0.6	0	0.6	**1**
Fever	11	13.3	16.4	15.8	**1**
Chills	1.4	2.4	2.7	3	**1**
Headache	5.5	3.6	9.6	7.3	**0.6**
Fatigue	16.4	12.7	28.8	20	**0.13**
Myalgia	5.5	1.2	5.5	2.4	**0.25**
Arthralgia	4.1	1.8	4.5	3	**0.7**
Generalized pruritus	0	0	0	0.6	**1**
Chest pain	0	0.6	0	1.2	**1**

*test for the second dose.

**Figure 3:**
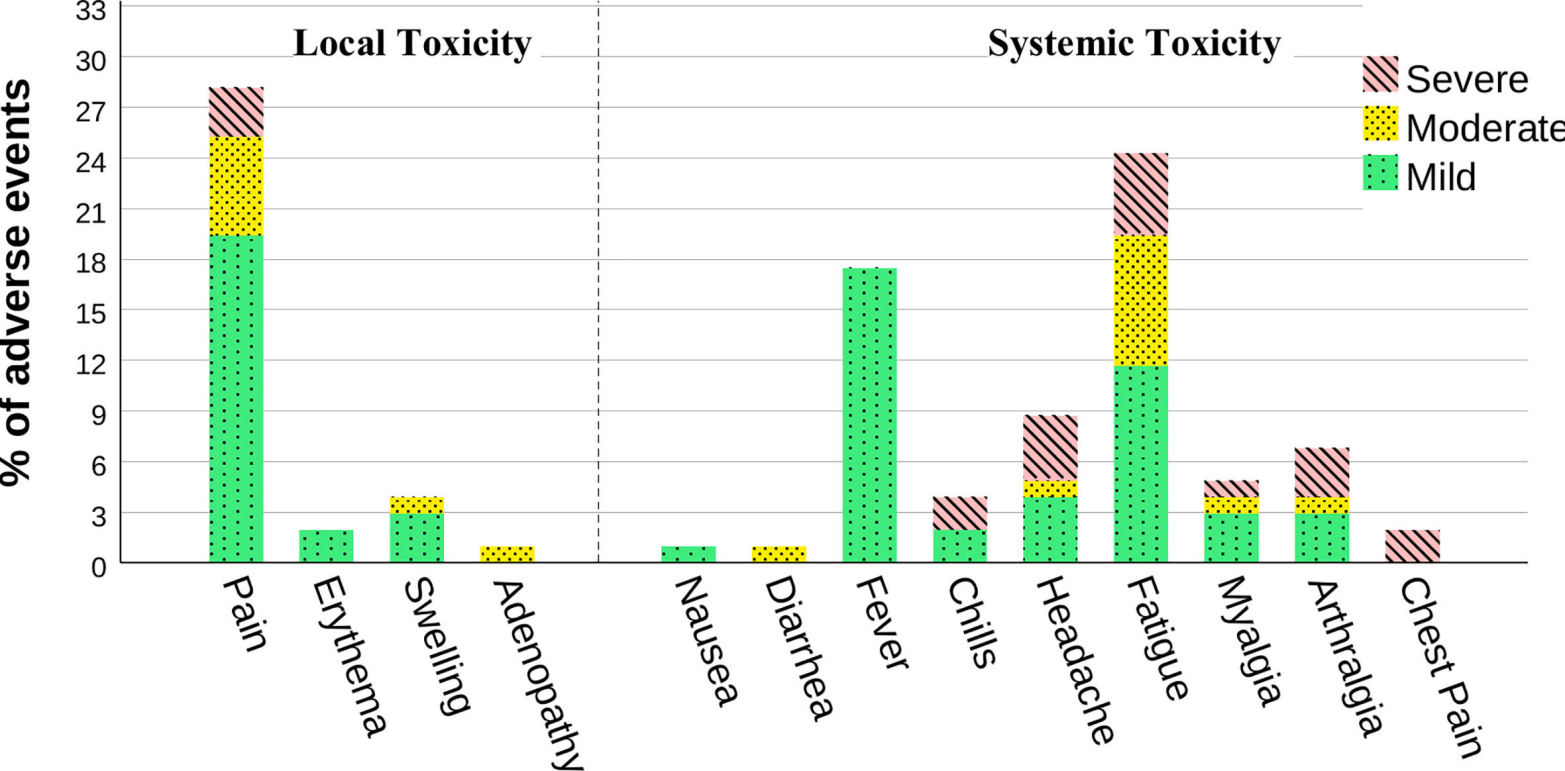
Adverse events reported after the second dose by cancer patients with active therapy.

In the adjusted subgroup analysis of vaccinated patients after the cancer diagnosis (n = 197) **Figure 3** communicates the frequency of adverse events following the second dose in people who were vaccinated while on active therapy (n = 103). With the Pearson’s chi-square independence test, no association between the prevalence of adverse events and active therapy was found (p=0.51, v= 0.04). While many patients under active therapy were to report more severe systemic toxicity that prevented daily activity (5.8% vs. 4.8%), using the Mann–Whitney U test, we observed no statistically significant difference in the distribution of severity grade of the symptoms reported after the first, as well as the second shot: local symptoms (p=0.52, r=0.04), systemic symptoms (p=0.22, r=0.09).

In our cohort, 93.6% (n = 280) of the patients who completed the initial protocol; had not yet received the booster dose: 43.6% declared they would not; because of fear of medical interactions, 10.7% under the influence of misleading reports in the media, 10.4% for medical reasons, 8.6% of fear of more severe adverse events compared to the first shots and finally, 26.7% declared their will to take the third shot 4 months after the second dose.

In this solid cancer population, nearly a quarter (n = 103, 24.8%) weren’t vaccinated: 35% refused the vaccination while receiving drug therapy, 14.5% were skeptical about the vaccine, 9.7% were due to medical recommendations, and finally one case of a pregnant woman.

## Discussion

Cancer patients receive an arsenal of treatments, therefore, they are considered a frail population. Reports about the mortality from COVID-19 among cancer patients were contradictory. While the first studies in China disclosed an increased risk of severe events (16), a large analysis in the United Kingdom stated that mortality was more linked to other clinical factors, rather than the therapy received (17). The extent to which the three pillars of cancer advancement have been impacted by the coronavirus must be emphasized. First, diverting pharma resources to face the pandemic caused a slowdown in cancer research (1). Second, according to studies, patients were less inclined to take part in clinical trials (18, 19). Last but not least, the effect of delay in treatment and diagnosis will affect the future prognosis of the disease (20). In the sight of this risk, the Moroccan Association for Training and Research in Medical Oncology, endorsed many measures, for instance, remote doctor-patient consultations; the use of oral drugs when possible; and rapid covid tests before chemotherapy sessions. And above all, insisted on vaccination as the best prophylactic means.

On the national level, four vaccines were granted for use with the subsequent protocols: Sinopharm vaccine: 28 days between doses, AstraZeneca vaccine: 21 days between doses, Pfizer vaccine: 23 days between doses, and Johnson & Johnson vaccine: -single dose-. The booster dose has been scheduled for all individuals four months after the last vaccine. The Sinopharm and AstraZeneca vaccines were early available; consequently, they were the most used for the standard scheme. The effectiveness and short-term safety of coronavirus vaccines have been demonstrated in numerous clinical trials. As a result, the Moroccan population achieved North Africa’s highest immunization rate (21, 22). Concerning the flu vaccination, the Moroccan strategy focused primarily on allocating resources to the COVID-19 vaccine, due to the initial concerns raised by a study in the United States regarding the coronavirus vaccination interfering with it (23).

To the best of our knowledge, this is the largest solid cancer cohort in North Africa that seeks to evaluate, 11 months after the launch of the vaccination campaign, the adverse events reported following the first, second and booster doses, and explore their association with the received drug therapy.

Indeed, in our pooled cohort, the vaccines used were generally well tolerated and no life-threatening events occurred. In line with prior studies on cancer patients (24–26), the most reported local toxicity was pain at the injection site. For the systemic effects, fatigue and fever were prevalent. Myalgia and arthralgia were also described in the context of flu-like symptoms. In our population, the rate of lymphadenopathy rose to 1.4%. Many studies highlighted the frequency of axillary nodes after covid vaccination, and the challenges caused in the oncology imaging (27–29). Even though lymph nodes could only be linked to cancer history, a prospective study of 232 cancer patients found an increase in regional nodes after COVID vaccination (30). Besides, we noted four cases of chest pain following the second shot. The same findings were communicated in a case series stating the incidence of myocarditis. However, for our patients, we couldn’t investigate the factual etiology (31, 32). In addition, we report a case of a male patient with generalized pruritus after immunization. Although urticaria reactions after covid vaccination are uncommon, they are well-documented in the literature (33, 34).

In another online cohort study; carried out in the United States, the authors found that the vaccine dose and brand were associated with the severity of adverse events (35). This report supports our finding that there were more events reported post second shot, and that the severity of systemic profiles after the second dose was significantly different from that, of the first one. However, our investigation was unable to show an association between the frequency of adverse events and the type of vaccine received.

In this real-world setting report, the toxicity profile and the cancer treatment received are independent. Furthermore, it was hypothesized that participants under active therapy reported more severe adverse events, than those with no active therapy. Our analyses, in contrast, did not support this hypothesis. These results were in agreement with a prospective study conducted in Iran; on 364 cancer patients vaccinated with the Sinopharm vaccine (36).

On the other hand, a meta-analysis of 35 studies suggested that patients undergoing cancer therapies were less likely to attain anti-SARS-CoV-2 spike protein (S) immunoglobulin G (IgG) seroconversion concentration rates after the standard immunization scheme compared to control groups (37), particularly in chemotherapy patients (38). This conclusion asserts the necessity of a booster dose in this population. Many factors, however, influence cancer patients’ choice to receive the covid vaccine. In a study conducted in Tunisia, educational level or history of comorbidities didn’t influence their adherence to the vaccination strategy (39). Yet, similar to our findings, their fear of interference with the cancer treatment or prognosis was a decisive factor. This lies behind the fact, that a quarter of our solid cancer population in Morocco was not immunized. These reasons could be extrapolated to the booster dose hesitancy too.

Our study has several strengths: the participants were randomly selected, and a large cohort was enrolled. For an accurate analysis, we divided the population into three sub-cohorts. Which allowed us to deepen the comparison between tumor patients vaccinated while receiving drug therapy and those who refused vaccination. Additionally, we adopted a semi-constructed interview to allow the participants to develop their answers, and thus minimize the biases.

As with the majority of studies, the current study’s design is subject to limitations. In this observational report, there was a potential recall bias that increased with the duration of data collection. In addition, most of our cancer population has not received the booster dose, so inferences about the toxicity profiles in this category couldn’t be made. Concerning some side effects, for instance, fatigue; nausea; and vomiting, their prevalence may have been biased by the cancer drugs received. Another limitation was the absence of a group vaccinated while on immunotherapy.

On the national level, no other local studies have been published to compare our current results. Nevertheless, the large size of our cohort may convey a representative view of the Moroccan solid cancer population. A natural progression of this work is to evaluate the side effects and adherence to the vaccination scheme, within patients at a hematologic malignancy center.

Many questions have emerged throughout the conducting of this study about the level of commitment to the vaccination protocol. Indeed, our findings brought to light the uncertainty and hesitancy expressed by the cancer population. On the other hand, and despite its exploratory nature, our report consolidates the existing data about the safety of the coronavirus vaccines in solid tumor patients. Hence, the challenge now is good communication between physicians, and their patients to assure adherence to the vaccination schemes. Which, given the potential of new outbreaks, will have a significant impact on public health plans for cancer prevention, screening, and treatment.

## Data availability statement

The raw data supporting the conclusions of this article will be made available by the authors, without undue reservation.

## Ethics statement

The study was reviewed and approved by the CERB: the biomedical ethics committee of the faculty of medicine of the University Mohammed V in Rabat-Morocco: Registered in Office for Human Research Protections under the IORG n°IORG0006594. The patients provided their written informed consent to participate in this study.

## Author contributions

HL and SB were involved in the conception of this study and the collection of data. HE was responsible for the oversight of the whole study and administrative support. HL analyzed the data. All authors interpreted and drafted the manuscript. All authors contributed to the article and approved the submitted version.

## Acknowledgments

The Cancer Research Institute provided access to the REDCap platform, which the authors gratefully acknowledge.

## Conflict of interest

The authors declare that the research was conducted in the absence of any commercial or financial relationships that could be construed as a potential conflict of interest.

## Publisher’s note

All claims expressed in this article are solely those of the authors and do not necessarily represent those of their affiliated organizations, or those of the publisher, the editors and the reviewers. Any product that may be evaluated in this article, or claim that may be made by its manufacturer, is not guaranteed or endorsed by the publisher.
