# Supplementary material for: Cancer patients and COVID-19 vaccination, from safety to protocol adherence: A real-life setting report
Source: Front Oncol. 2022 Oct 3;12:1014786. doi: 10.3389/fonc.2022.1014786 (PMC9573984; doi:10.3389/fonc.2022.1014786)
Supplement: Supplementary file 1 [file Table_1.docx]

| Comorbidities | | Vaccine before cancer dg  n=115 | Vaccine after cancer dg  n=197 | Not vaccinated  n=103 |
| --- | --- | --- | --- | --- |
| High blood pressure | | 29 | 23 | 17 |
| Congestive Heart Failure | | 0 | 3 | 1 |
| Peripheral vascular disease | | 4 | 2 | 2 |
| CVA or TIA^1^ | | 0 | 1 | 1 |
| Dementia | | 1 | 2 | 2 |
| Chronic lung disease | | 23 | 36 | 13 |
| Rheumatic or Connective tissue disease | | 4 | 1 | 0 |
| Peptic ulcer disease | | 1 | 0 | 0 |
| Liver Disease | Mild | 1 | 2 | 0 |
|  | Moderate to severe | 1 | 2 | 0 |
| Diabetes mellitus | Uncomplicated | 15 | 10 | 6 |
|  | End-organ damage | 6 | 6 | 8 |
| Hemiplegia or paraplegia | | 1 | 0 | 2 |
| Chronic Kidney disease | | 3 | 3 | 1 |
| Solid Tumor | Localized | 63 | 77 | 42 |
|  | Metastatic | 52 | 120 | 61 |
| AIDS | | 0 | 0 | 1 |

**Supplementary table S1. Detailed comorbidities in our cohort.**

^1^: Cerebrovascular accident or Transient ischemic attack
