# Supplementary material for: Cancer patients and COVID-19 vaccination, from safety to protocol adherence: A real-life setting report
Source: Front Oncol. 2022 Oct 3;12:1014786. doi: 10.3389/fonc.2022.1014786 (PMC9573984; doi:10.3389/fonc.2022.1014786)
Supplement: Supplementary file 2 [file Table_2.docx]

|  | | Toxicity after 2^nd^ dose | | Total |
| --- | --- | --- | --- | --- |
|  |  | No | Yes |  |
| Toxicity after  1^st^ dose | No | 138 | 48 | 186 |
|  | Yes | 24 | 86 | 110 |
| Total | | 162 | 134 | 296 |

**Supplementary table S2.** **Consistency in responses of patients who answered questions about the symptoms after the first and second doses.**
